# Supplementary material for: Deletion of wfs1 Impairs Oligodendrocyte Precursor Cells Dorsal Distribution and Myelination Through the wfs1-hmgcs1 Axis in Zebrafish
Source: Biology (Basel). 2026 Aug 16;15(16):1402. doi: 10.3390/biology15161402 (PMC13510151; doi:10.3390/biology15161402)
Supplement: Supplementary file 1 [file biology-15-01402-s001.zip › File S1-Transcriptome sequencing data and differentially expressed genes (DEGs) in wfs1-- zebrafish at 5 dpf/2.KEGG/wfs1KOvsWT/GSEA/WT/WNT_SIGNALING_PATHWAY/WNT_SIGNALING_PATHWAY(DRE04310).html]

Details for gene set WNT\_SIGNALING\_PATHWAY(DRE04310)[GSEA]

|  || Dataset | deg.sample |
| Phenotype | sample.cls |
| Upregulated in class | WT |
| GeneSet | WNT\_SIGNALING\_PATHWAY(DRE04310) |
| Enrichment Score (ES) | -0.21183707 |
| Normalized Enrichment Score (NES) | -1.0197147 |
| Nominal p-value | 0.31771895 |
| FDR q-value | 0.56610906 |
| FWER p-Value | 1.0 |
Table: GSEA Results Summary

  

Fig 1: Enrichment plot: WNT\_SIGNALING\_PATHWAY(DRE04310)      
 Profile of the Running ES Score & Positions of GeneSet Members on the Rank Ordered List

  

| PROBE | DESCRIPTION (from dataset) | GENE SYMBOL | GENE\_TITLE | RANK IN GENE LIST | RANK METRIC SCORE | RUNNING ES | CORE ENRICHMENT || 1 | 566841 | crebbpa |  |  | 530 | 2.904 | 0.0027 | No |
| 2 | 100000500\_1 | sost |  |  | 681 | 2.622 | 0.0155 | No |
| 3 | 100006675 | prkcba |  |  | 711 | 2.563 | 0.0317 | No |
| 4 | 436718 | rhocb |  |  | 948 | 2.171 | 0.0388 | No |
| 5 | 678649\_1 | zgc:136929 |  |  | 1161 | 1.938 | 0.0450 | No |
| 6 | 58072\_1 | fzd8b |  |  | 1186 | 1.912 | 0.0571 | No |
| 7 | 556087 | wnt2bb |  |  | 1255 | 1.847 | 0.0673 | No |
| 8 | 103909653 | LOC103909653 |  |  | 1406 | 1.721 | 0.0741 | No |
| 9 | 100006275 | porcn |  |  | 1585 | 1.602 | 0.0792 | No |
| 10 | 101882911 | LOC101882911 |  |  | 1827 | 1.446 | 0.0812 | No |
| 11 | 564241 | fosl1a |  |  | 2306 | 1.247 | 0.0744 | No |
| 12 | 58023\_1 | fzd9b |  |  | 2350 | 1.227 | 0.0813 | No |
| 13 | 359837 | wnt2ba |  |  | 2798 | 1.084 | 0.0744 | No |
| 14 | 323883\_1 | cul1a |  |  | 2816 | 1.080 | 0.0811 | No |
| 15 | 101884483 | LOC101884483 |  |  | 2886 | 1.061 | 0.0860 | No |
| 16 | 30590\_1 | tp53 |  |  | 2944 | 1.051 | 0.0913 | No |
| 17 | 791993\_1 | wnt4b |  |  | 2954 | 1.048 | 0.0980 | No |
| 18 | 30206 | tle3a |  |  | 3032 | 1.028 | 0.1024 | No |
| 19 | 565038 | ccnd2b |  |  | 3069 | 1.021 | 0.1081 | No |
| 20 | 791563 | wnt16 |  |  | 3185 | 0.995 | 0.1111 | No |
| 21 | 326875 | cby1 |  |  | 3462 | 0.938 | 0.1087 | No |
| 22 | 565241 | ppp3ccb |  |  | 3535 | 0.922 | 0.1126 | No |
| 23 | 100001408 | apcdd1l |  |  | 3668 | 0.898 | 0.1144 | No |
| 24 | 58073 | fzd8a |  |  | 3761 | 0.876 | 0.1174 | No |
| 25 | 447814 | ppp3r1b |  |  | 3939 | 0.841 | 0.1174 | No |
| 26 | 100333254 | nfatc2a |  |  | 4008 | 0.829 | 0.1208 | No |
| 27 | 30105 | wnt5b |  |  | 4244 | 0.787 | 0.1186 | No |
| 28 | 564019 | si:dkey-42p8.3 |  |  | 4418 | 0.757 | 0.1182 | No |
| 29 | 794221 | nkd2b |  |  | 4454 | 0.751 | 0.1222 | No |
| 30 | 368485\_2 | ctnnd2a |  |  | 4526 | 0.740 | 0.1249 | No |
| 31 | 793648 | LOC793648 |  |  | 4799 | 0.705 | 0.1210 | No |
| 32 | 751630 | zgc:153713 |  |  | 4826 | 0.702 | 0.1249 | No |
| 33 | 100537538 | LOC100537538 |  |  | 4897 | 0.694 | 0.1273 | No |
| 34 | 100148840 | wnt7bb |  |  | 5854 | 0.602 | 0.1011 | No |
| 35 | 100006858 | csnk1e |  |  | 5879 | 0.599 | 0.1043 | No |
| 36 | 791595\_1 | wnt11 |  |  | 5972 | 0.590 | 0.1054 | No |
| 37 | 393998 | prkacba |  |  | 6234 | 0.564 | 0.1009 | No |
| 38 | 30719 | gbp |  |  | 6298 | 0.557 | 0.1026 | No |
| 39 | 58092\_1 | smad3a |  |  | 6307 | 0.555 | 0.1061 | No |
| 40 | 561772 | nfatc3a |  |  | 6357 | 0.552 | 0.1082 | No |
| 41 | 30171 | wnt10a |  |  | 6358 | 0.551 | 0.1119 | No |
| 42 | 557384 | fzd1 |  |  | 6390 | 0.548 | 0.1146 | No |
| 43 | 798222 | camk2b |  |  | 6413 | 0.544 | 0.1176 | No |
| 44 | 565241\_1 | ppp3ccb |  |  | 6607 | 0.527 | 0.1150 | No |
| 45 | 101884425 | LOC101884425 |  |  | 6612 | 0.527 | 0.1184 | No |
| 46 | 559273\_1 | ep300a |  |  | 6707 | 0.519 | 0.1189 | No |
| 47 | 436625 | rspo1 |  |  | 6790 | 0.511 | 0.1197 | No |
| 48 | 30544 | sox17 |  |  | 6815 | 0.509 | 0.1223 | No |
| 49 | 30654 | gsk3b |  |  | 6859 | 0.504 | 0.1244 | No |
| 50 | 30686 | myca |  |  | 6877 | 0.502 | 0.1272 | No |
| 51 | 791563\_1 | wnt16 |  |  | 6996 | 0.492 | 0.1267 | No |
| 52 | 65236 | mapk8b |  |  | 6998 | 0.492 | 0.1300 | No |
| 53 | 30127 | wnt2 |  |  | 7041 | 0.488 | 0.1319 | No |
| 54 | 557926\_2 | ppp3cca |  |  | 7193 | 0.474 | 0.1303 | No |
| 55 | 335916 | jun |  |  | 7293 | 0.467 | 0.1303 | No |
| 56 | 100151181 | si:dkey-239n17.4 |  |  | 7340 | 0.463 | 0.1320 | No |
| 57 | 556813 | znrf3 |  |  | 7423 | 0.459 | 0.1325 | No |
| 58 | 103909610 | LOC103909610 |  |  | 7518 | 0.453 | 0.1325 | No |
| 59 | 793359\_1 | camk2g2 |  |  | 7602 | 0.447 | 0.1329 | No |
| 60 | 568214 | chd8 |  |  | 7705 | 0.440 | 0.1326 | No |
| 61 | 368250 | prickle2b |  |  | 7881 | 0.432 | 0.1300 | No |
| 62 | 100006132 | si:ch211-1f22.2 |  |  | 8076 | 0.417 | 0.1266 | No |
| 63 | 751644 | wnt9a |  |  | 8426 | 0.408 | 0.1183 | No |
| 64 | 561772\_1 | nfatc3a |  |  | 8493 | 0.403 | 0.1189 | No |
| 65 | 566869 | nfatc3b |  |  | 8542 | 0.399 | 0.1201 | No |
| 66 | 557926 | ppp3cca |  |  | 8563 | 0.398 | 0.1221 | No |
| 67 | 100538205 | sfrp2l |  |  | 8726 | 0.386 | 0.1196 | No |
| 68 | 192307\_1 | csnk1a1 |  |  | 9214 | 0.352 | 0.1065 | No |
| 69 | 415254 | zgc:86598 |  |  | 9372 | 0.343 | 0.1038 | No |
| 70 | 553788 | map3k7 |  |  | 9427 | 0.338 | 0.1044 | No |
| 71 | 792404 | dkk2 |  |  | 9465 | 0.336 | 0.1055 | No |
| 72 | 100332639 | LOC100332639 |  |  | 9525 | 0.332 | 0.1058 | No |
| 73 | 793359 | camk2g2 |  |  | 9631 | 0.325 | 0.1047 | No |
| 74 | 30556 | tcf7l1b |  |  | 9704 | 0.321 | 0.1046 | No |
| 75 | 30366 | fzd10 |  |  | 9743 | 0.319 | 0.1055 | No |
| 76 | 436917 | notum1b |  |  | 9772 | 0.317 | 0.1067 | No |
| 77 | 562720 | mapk8a |  |  | 9888 | 0.310 | 0.1052 | No |
| 78 | 245949 | vangl2 |  |  | 9919 | 0.309 | 0.1063 | No |
| 79 | 566040 | notum2 |  |  | 9963 | 0.305 | 0.1070 | No |
| 80 | 560317 | si:dkey-222b8.1 |  |  | 10062 | 0.300 | 0.1059 | No |
| 81 | 323883\_2 | cul1a |  |  | 10317 | 0.284 | 0.0997 | No |
| 82 | 30523 | tcf7l1a |  |  | 10339 | 0.282 | 0.1010 | No |
| 83 | 393141 | mycb |  |  | 10475 | 0.274 | 0.0985 | No |
| 84 | 568282 | ror1 |  |  | 10693 | 0.261 | 0.0934 | No |
| 85 | 30364 | fzd5 |  |  | 10873 | 0.250 | 0.0894 | No |
| 86 | 497384 | prkca |  |  | 10900 | 0.249 | 0.0903 | No |
| 87 | 553608 | tbl1xr1a |  |  | 10916 | 0.248 | 0.0915 | No |
| 88 | 100331321 | LOC100331321 |  |  | 10929 | 0.248 | 0.0927 | No |
| 89 | 407700 | plcb4 |  |  | 11151 | 0.236 | 0.0873 | No |
| 90 | 100149080 | LOC100149080 |  |  | 11197 | 0.233 | 0.0875 | No |
| 91 | 565714 | wnt7aa |  |  | 11249 | 0.231 | 0.0874 | No |
| 92 | 30221 | psen1 |  |  | 11456 | 0.219 | 0.0823 | No |
| 93 | 497649 | tcf7 |  |  | 11529 | 0.215 | 0.0815 | No |
| 94 | 326283 | smad3b |  |  | 11759 | 0.203 | 0.0756 | No |
| 95 | 393953 | prkcbb |  |  | 11901 | 0.196 | 0.0725 | No |
| 96 | 58117 | ctnnbip1 |  |  | 12016 | 0.190 | 0.0701 | No |
| 97 | 100003514 | prkcg |  |  | 12176 | 0.181 | 0.0663 | No |
| 98 | 117510 | sfrp5 |  |  | 12185 | 0.180 | 0.0673 | No |
| 99 | 317747 | rock2a |  |  | 12313 | 0.173 | 0.0644 | No |
| 100 | 403076 | vangl1 |  |  | 12332 | 0.172 | 0.0650 | No |
| 101 | 30510\_1 | tcf7l2 |  |  | 12361 | 0.171 | 0.0652 | No |
| 102 | 794669 | gb:cr929477 |  |  | 12490 | 0.165 | 0.0623 | No |
| 103 | 561682 | nfatc4 |  |  | 12560 | 0.162 | 0.0612 | No |
| 104 | 568518 | lrp5 |  |  | 12728 | 0.152 | 0.0569 | No |
| 105 | 368249 | prickle1a |  |  | 12902 | 0.145 | 0.0524 | No |
| 106 | 100003877 | lrp6 |  |  | 12998 | 0.140 | 0.0504 | No |
| 107 | 566878\_1 | sfrp2 |  |  | 13021 | 0.139 | 0.0506 | No |
| 108 | 556400 | senp2 |  |  | 13068 | 0.136 | 0.0501 | No |
| 109 | 562064 | camk2b1 |  |  | 13117 | 0.134 | 0.0494 | No |
| 110 | 100330212 | lgr6 |  |  | 13417 | 0.120 | 0.0408 | No |
| 111 | 569863 | dvl1a |  |  | 13418 | 0.120 | 0.0416 | No |
| 112 | 567111 | crebbpb |  |  | 13470 | 0.117 | 0.0407 | No |
| 113 | 560294 | tbl1x |  |  | 13664 | 0.108 | 0.0354 | No |
| 114 | 393533 | fzd6 |  |  | 13710 | 0.106 | 0.0347 | No |
| 115 | 569698 | mapk10 |  |  | 13907 | 0.096 | 0.0291 | No |
| 116 | 563147 | prkacbb |  |  | 13940 | 0.095 | 0.0287 | No |
| 117 | 798465 | daam2 |  |  | 14192 | 0.083 | 0.0213 | No |
| 118 | 368485\_1 | ctnnd2a |  |  | 14269 | 0.079 | 0.0195 | No |
| 119 | 100003877\_1 | lrp6 |  |  | 14274 | 0.079 | 0.0199 | No |
| 120 | 556379 | csnk2a2b |  |  | 14295 | 0.078 | 0.0198 | No |
| 121 | 30370 | fzd2 |  |  | 14334 | 0.077 | 0.0191 | No |
| 122 | 557451 | daam1a |  |  | 14389 | 0.075 | 0.0179 | No |
| 123 | 393716 | skp1 |  |  | 14456 | 0.071 | 0.0163 | No |
| 124 | 564064 | prkacaa |  |  | 14469 | 0.071 | 0.0164 | No |
| 125 | 100001532 | plcb3 |  |  | 14510 | 0.069 | 0.0155 | No |
| 126 | 30701 | lef1 |  |  | 14523 | 0.068 | 0.0156 | No |
| 127 | 447800 | serpinf1 |  |  | 14532 | 0.068 | 0.0158 | No |
| 128 | 57931 | axin1 |  |  | 14585 | 0.066 | 0.0146 | No |
| 129 | 100007463 | tle3b |  |  | 14593 | 0.065 | 0.0148 | No |
| 130 | 118437 | gpc4 |  |  | 14777 | 0.057 | 0.0094 | No |
| 131 | 792714 | wisp1b |  |  | 15069 | 0.043 | 0.0005 | No |
| 132 | 565612 | ep300b |  |  | 15569 | 0.019 | -0.0152 | No |
| 133 | 65229\_1 | ctbp1 |  |  | 15700 | 0.012 | -0.0192 | No |
| 134 | 799608 | ccnd2a |  |  | 15743 | 0.009 | -0.0205 | No |
| 135 | 569420 | wnt3 |  |  | 15807 | 0.006 | -0.0224 | No |
| 136 | 558502 | ryk |  |  | 15842 | 0.005 | -0.0234 | No |
| 137 | 565921 | fzd3a |  |  | 15924 | 0.001 | -0.0260 | No |
| 138 | 101884148\_1 | LOC101884148 |  |  | 15965 | -0.000 | -0.0273 | No |
| 139 | 557447 | fzd9a |  |  | 16011 | -0.002 | -0.0287 | No |
| 140 | 30754 | ppardb |  |  | 16357 | -0.013 | -0.0395 | No |
| 141 | 559111 | smad4a |  |  | 16436 | -0.016 | -0.0419 | No |
| 142 | 80972 | dvl3a |  |  | 16463 | -0.016 | -0.0426 | No |
| 143 | 563460 | nlk2 |  |  | 16472 | -0.017 | -0.0427 | No |
| 144 | 321070 | rock2b |  |  | 16494 | -0.017 | -0.0433 | No |
| 145 | 368485 | ctnnd2a |  |  | 16773 | -0.025 | -0.0519 | No |
| 146 | 559273 | ep300a |  |  | 16774 | -0.025 | -0.0517 | No |
| 147 | 795167 | rac3b |  |  | 17171 | -0.040 | -0.0640 | No |
| 148 | 445076 | prkacab |  |  | 17201 | -0.041 | -0.0646 | No |
| 149 | 30510 | tcf7l2 |  |  | 17360 | -0.048 | -0.0693 | No |
| 150 | 30412 | fzd7b |  |  | 17559 | -0.056 | -0.0752 | No |
| 151 | 30363 | fzd4 |  |  | 17601 | -0.058 | -0.0761 | No |
| 152 | 560032 | dvl1b |  |  | 17603 | -0.058 | -0.0757 | No |
| 153 | 324004 | ctnnb2 |  |  | 17720 | -0.064 | -0.0790 | No |
| 154 | 100149273 | mapk9 |  |  | 17865 | -0.070 | -0.0831 | No |
| 155 | 100330136 | LOC100330136 |  |  | 17867 | -0.070 | -0.0826 | No |
| 156 | 405892 | nlk1 |  |  | 17881 | -0.071 | -0.0826 | No |
| 157 | 30119 | frzb |  |  | 18029 | -0.077 | -0.0867 | No |
| 158 | 386762 | apc |  |  | 18144 | -0.083 | -0.0898 | No |
| 159 | 406411 | rhoaa |  |  | 18330 | -0.091 | -0.0950 | No |
| 160 | 101883172 | LOC101883172 |  |  | 18491 | -0.098 | -0.0994 | No |
| 161 | 80955 | siah1 |  |  | 18548 | -0.101 | -0.1005 | No |
| 162 | 30265 | ctnnb1 |  |  | 18549 | -0.101 | -0.0998 | No |
| 163 | 570116 | prickle3 |  |  | 18645 | -0.105 | -0.1021 | No |
| 164 | 567746 | fzd3b |  |  | 18726 | -0.110 | -0.1039 | No |
| 165 | 245946 | invs |  |  | 18882 | -0.117 | -0.1080 | No |
| 166 | 777736 | prickle2a |  |  | 18930 | -0.119 | -0.1087 | No |
| 167 | 751108 | nkd1 |  |  | 18935 | -0.119 | -0.1081 | No |
| 168 | 327204 | rac1a |  |  | 19053 | -0.124 | -0.1109 | No |
| 169 | 559273\_2 | ep300a |  |  | 19072 | -0.125 | -0.1107 | No |
| 170 | 30488 | csnk2a2a |  |  | 19129 | -0.128 | -0.1116 | No |
| 171 | 323630 | dvl2 |  |  | 19187 | -0.130 | -0.1125 | No |
| 172 | 560713 | wisp1a |  |  | 19198 | -0.131 | -0.1120 | No |
| 173 | 560060 | daam1b |  |  | 19289 | -0.135 | -0.1139 | No |
| 174 | 566054\_1 | rspo2 |  |  | 19513 | -0.144 | -0.1200 | No |
| 175 | 565677 | wnt9b |  |  | 19640 | -0.150 | -0.1230 | No |
| 176 | 30502 | csnk2a1 |  |  | 19706 | -0.153 | -0.1240 | No |
| 177 | 571865 | LOC571865 |  |  | 19757 | -0.155 | -0.1245 | No |
| 178 | 30428 | csnk2b |  |  | 19758 | -0.155 | -0.1235 | No |
| 179 | 558825 | lgr4 |  |  | 19990 | -0.166 | -0.1297 | No |
| 180 | 572332 | rnf43 |  |  | 20204 | -0.175 | -0.1353 | No |
| 181 | 791855 | camk2g1 |  |  | 20264 | -0.177 | -0.1360 | No |
| 182 | 565013 | tle2 |  |  | 20348 | -0.181 | -0.1374 | No |
| 183 | 569376 | plcb2 |  |  | 20503 | -0.188 | -0.1410 | No |
| 184 | 65230 | ctbp2a |  |  | 20504 | -0.188 | -0.1397 | No |
| 185 | 405823 | cacybp |  |  | 20582 | -0.193 | -0.1409 | No |
| 186 | 794574 | ppp3ca |  |  | 20620 | -0.195 | -0.1407 | No |
| 187 | 406816 | cul1b |  |  | 20667 | -0.197 | -0.1409 | No |
| 188 | 567111\_1 | crebbpb |  |  | 20683 | -0.198 | -0.1400 | No |
| 189 | 556148 | prickle1b |  |  | 20962 | -0.214 | -0.1474 | No |
| 190 | 100535532 | csnk2a4 |  |  | 20986 | -0.215 | -0.1467 | No |
| 191 | 568315 | nfatc1 |  |  | 20990 | -0.215 | -0.1453 | No |
| 192 | 100006754 | cxxc4 |  |  | 21106 | -0.221 | -0.1475 | No |
| 193 | 550554 | ppp3cb |  |  | 21144 | -0.223 | -0.1472 | No |
| 194 | 30128 | wnt1 |  |  | 21411 | -0.236 | -0.1540 | No |
| 195 | 333999 | porcnl |  |  | 21460 | -0.238 | -0.1539 | No |
| 196 | 30222 | ccnd1 |  |  | 21522 | -0.242 | -0.1542 | No |
| 197 | 548605 | ctbp2l |  |  | 21883 | -0.261 | -0.1639 | No |
| 198 | 30283 | wnt11r |  |  | 21916 | -0.263 | -0.1631 | No |
| 199 | 334373 | fbxw11a |  |  | 22046 | -0.270 | -0.1654 | No |
| 200 | 406825 | fbxw11b |  |  | 22332 | -0.286 | -0.1725 | No |
| 201 | 550436 | camk2a |  |  | 22386 | -0.289 | -0.1722 | No |
| 202 | 58080 | axin2 |  |  | 22397 | -0.290 | -0.1706 | No |
| 203 | 58092 | smad3a |  |  | 22399 | -0.290 | -0.1687 | No |
| 204 | 317679 | ruvbl1 |  |  | 22437 | -0.292 | -0.1679 | No |
| 205 | 100332722 | wnt6a |  |  | 22571 | -0.300 | -0.1701 | No |
| 206 | 100007702 | rspo3 |  |  | 22690 | -0.307 | -0.1718 | No |
| 207 | 799377 | dkk1a |  |  | 22721 | -0.309 | -0.1707 | No |
| 208 | 558069 | ctnnd2b |  |  | 22958 | -0.324 | -0.1760 | No |
| 209 | 570510 | notum1a |  |  | 23010 | -0.328 | -0.1754 | No |
| 210 | 30144 | wnt8b |  |  | 23062 | -0.332 | -0.1748 | No |
| 211 | 406659 | rhoac |  |  | 23154 | -0.338 | -0.1754 | No |
| 212 | 431736 | bambib |  |  | 23399 | -0.354 | -0.1808 | No |
| 213 | 553231 | ccdc88c |  |  | 23484 | -0.360 | -0.1810 | No |
| 214 | 557882 | si:dkeyp-80c12.7 |  |  | 23526 | -0.363 | -0.1799 | No |
| 215 | 548605\_1 | ctbp2l |  |  | 23705 | -0.373 | -0.1830 | No |
| 216 | 563427 | wnt7ba |  |  | 23760 | -0.377 | -0.1822 | No |
| 217 | 565771 | apc2 |  |  | 23827 | -0.382 | -0.1817 | No |
| 218 | 561183 | ror2 |  |  | 23881 | -0.385 | -0.1808 | No |
| 219 | 561831 | wnt6b |  |  | 24096 | -0.399 | -0.1849 | No |
| 220 | 30750 | pparda |  |  | 24113 | -0.400 | -0.1828 | No |
| 221 | 568214\_1 | chd8 |  |  | 24128 | -0.401 | -0.1805 | No |
| 222 | 794669\_1 | gb:cr929477 |  |  | 24180 | -0.405 | -0.1794 | No |
| 223 | 566054 | rspo2 |  |  | 24232 | -0.409 | -0.1783 | No |
| 224 | 565013\_1 | tle2 |  |  | 24234 | -0.409 | -0.1756 | No |
| 225 | 101884148 | LOC101884148 |  |  | 24339 | -0.415 | -0.1761 | No |
| 226 | 402843 | sfrp1a |  |  | 24767 | -0.436 | -0.1867 | No |
| 227 | 100006041 | rhoab |  |  | 24773 | -0.436 | -0.1839 | No |
| 228 | 449846 | rbx1 |  |  | 24797 | -0.438 | -0.1817 | No |
| 229 | novel.818 | - |  |  | 25208 | -0.460 | -0.1916 | No |
| 230 | 101886083 | LOC101886083 |  |  | 25338 | -0.469 | -0.1925 | No |
| 231 | 568191 | wnt5a |  |  | 25472 | -0.482 | -0.1935 | No |
| 232 | 30476 | wif1 |  |  | 25579 | -0.492 | -0.1936 | No |
| 233 | 393581 | tbl1xr1b |  |  | 25656 | -0.499 | -0.1927 | No |
| 234 | 100006275\_1 | porcn |  |  | 25657 | -0.499 | -0.1893 | No |
| 235 | 568110 | LOC568110 |  |  | 25683 | -0.501 | -0.1868 | No |
| 236 | 436815 | camk2d2 |  |  | 25880 | -0.518 | -0.1895 | No |
| 237 | 30308 | wnt10b |  |  | 26044 | -0.533 | -0.1911 | No |
| 238 | 83496 | bambia |  |  | 26470 | -0.575 | -0.2007 | No |
| 239 | 100333967 | si:ch73-374l24.1 |  |  | 26564 | -0.584 | -0.1997 | No |
| 240 | 437027 | rac3a |  |  | 26579 | -0.586 | -0.1962 | No |
| 241 | 100149082 | dvl3 |  |  | 26821 | -0.611 | -0.1998 | No |
| 242 | 569420\_1 | wnt3 |  |  | 26844 | -0.613 | -0.1963 | No |
| 243 | 445208 | camk2d1 |  |  | 26863 | -0.615 | -0.1928 | No |
| 244 | 566878 | sfrp2 |  |  | 27093 | -0.639 | -0.1958 | No |
| 245 | 415151 | rac2 |  |  | 27490 | -0.672 | -0.2038 | No |
| 246 | 30123 | wnt4a |  |  | 27492 | -0.672 | -0.1993 | No |
| 247 | 449996 | ccnd3 |  |  | 27759 | -0.710 | -0.2030 | No |
| 248 | 497384\_1 | prkca |  |  | 27854 | -0.723 | -0.2011 | No |
| 249 | 60632 | wnt3a |  |  | 28059 | -0.750 | -0.2026 | No |
| 250 | 110438182 | LOC110438182 |  |  | 28145 | -0.763 | -0.2001 | No |
| 251 | 562838 | rac1b |  |  | 28146 | -0.763 | -0.1950 | No |
| 252 | 65229 | ctbp1 |  |  | 28678 | -0.852 | -0.2061 | Yes |
| 253 | 30206\_1 | tle3a |  |  | 28710 | -0.860 | -0.2014 | Yes |
| 254 | 100006208 | LOC100006208 |  |  | 28738 | -0.866 | -0.1964 | Yes |
| 255 | 100006166 | si:ch211-1f22.1 |  |  | 28986 | -0.917 | -0.1981 | Yes |
| 256 | 100001408\_1 | apcdd1l |  |  | 29185 | -0.970 | -0.1979 | Yes |
| 257 | 101884327 | LOC101884327 |  |  | 29590 | -1.090 | -0.2033 | Yes |
| 258 | 564337 | ppp3r1a |  |  | 29604 | -1.097 | -0.1964 | Yes |
| 259 | 30197 | dkk1b |  |  | 29636 | -1.107 | -0.1900 | Yes |
| 260 | 792699 | rac1l |  |  | 30004 | -1.247 | -0.1932 | Yes |
| 261 | 798564 | sfrp1b |  |  | 30070 | -1.272 | -0.1868 | Yes |
| 262 | 100006675\_1 | prkcba |  |  | 30078 | -1.276 | -0.1785 | Yes |
| 263 | 100006858\_1 | csnk1e |  |  | 30290 | -1.385 | -0.1759 | Yes |
| 264 | 30590 | tp53 |  |  | 30325 | -1.401 | -0.1676 | Yes |
| 265 | 100000500 | sost |  |  | 30445 | -1.474 | -0.1615 | Yes |
| 266 | 566841\_1 | crebbpa |  |  | 30571 | -1.563 | -0.1550 | Yes |
| 267 | 402843\_1 | sfrp1a |  |  | 30678 | -1.651 | -0.1473 | Yes |
| 268 | 101883269 | LOC101883269 |  |  | 30732 | -1.709 | -0.1375 | Yes |
| 269 | 791993 | wnt4b |  |  | 30739 | -1.714 | -0.1262 | Yes |
| 270 | 326875\_1 | cby1 |  |  | 30767 | -1.732 | -0.1155 | Yes |
| 271 | 30369\_1 | fzd7a |  |  | 30807 | -1.766 | -0.1049 | Yes |
| 272 | 30127\_1 | wnt2 |  |  | 30890 | -1.852 | -0.0951 | Yes |
| 273 | 323883 | cul1a |  |  | 31083 | -2.063 | -0.0874 | Yes |
| 274 | 678649 | zgc:136929 |  |  | 31464 | -2.814 | -0.0806 | Yes |
| 275 | 58072 | fzd8b |  |  | 31520 | -2.978 | -0.0624 | Yes |
| 276 | 565241\_2 | ppp3ccb |  |  | 31577 | -3.181 | -0.0428 | Yes |
| 277 | 556087\_1 | wnt2bb |  |  | 31672 | -3.576 | -0.0219 | Yes |
| 278 | 436718\_1 | rhocb |  |  | 31770 | -4.295 | 0.0038 | Yes |
Table: GSEA details [plain text format]

  

Fig 2: WNT\_SIGNALING\_PATHWAY(DRE04310)      
 Blue-Pink O' Gram in the Space of the Analyzed GeneSet

  

Fig 3: WNT\_SIGNALING\_PATHWAY(DRE04310): Random ES distribution      
 Gene set null distribution of ES for **WNT\_SIGNALING\_PATHWAY(DRE04310)**
